# Adsorption and Removal of Cr^6+^, Cu^2+^, Pb^2+^, and Zn^2+^ from Aqueous Solution by Magnetic Nano-Chitosan

**DOI:** 10.3390/molecules28062607

**Published:** 2023-03-13

**Authors:** Yuran He, Panqing Zhang, Lijun Wang

**Affiliations:** Department of Environmental Science and Engineering, School of Geography and Tourism, Shaanxi Normal University, Xi’an 710119, China; heyuran@snnu.edu.cn (Y.H.); zhangpanqing123@163.com (P.Z.)

**Keywords:** magnetic nano-chitosan, preparation and characterization, heavy metal ion, adsorption removal

## Abstract

Magnetic nano-chitosan (MNC) was prepared and characterized. The kinetics, thermodynamics, and influencing factors of the adsorption of Cr^6+^, Cu^2+^, Pb^2+^, and Zn^2+^, as well as their competitive adsorption onto MNC in aqueous solution, were studied. The results showed that the adsorption kinetics and thermodynamics of Cr^6+^, Cu^2+^, Pb^2+^, and Zn^2+^ were well described by the pseudo-second-order kinetic model and Langmuir isothermal adsorption model, indicating that the adsorption was mainly chemical adsorption and endothermic. Increasing the dosage of MNC, the equilibrium adsorption capacity (q_e_) of Cr^6+^, Cu^2+^, Pb^2+^, and Zn^2+^ decreased; their removal rate (η) increased. With the increase in the solution’s pH, the q_e_ and η of Cr^6+^ first increased and then decreased; the q_e_ and η of Cu^2+^, Pb^2+^, and Zn^2+^ increased. With the increase in the metal ion initial concentration, the q_e_ increased; the η of Cr^6+^, Cu^2+^, and Zn^2+^ decreased, while the η of Pb^2+^ increased first and then decreased. Temperature had a weak influence on the q_e_ of Cr^6+^ and Pb^2+^, while it had a strong influence on Cu^2+^ and Zn^2+^, the q_e_ and η were greater when the temperature was higher, and the adsorption was spontaneous and endothermic. The q_e_ and η of Cu^2+^, Pb^2+^, and Zn^2+^ decreased in the presence of co-existing ions. The influences among metal ions existed in a binary and ternary ion system. The current study’s results provide a theoretical support for the simultaneous treatment of harmful metal ions in wastewater by MNC.

## 1. Introduction

Water is one of our most important resources. However, various anthropogenic activities, such as mining, metal smelting, electroplating, and leather, have caused the severe pollution of water by heavy metals, which has received extensive concern around the world [1,2,3]. Heavy metals are characterized by potential bio-accumulation, non-biodegradation, and high toxicity, seriously affecting ecological environment quality and human health [4,5,6,7,8,9]. The common heavy metals in water are Cr, Cu, Pb, Zn, Hg, Cd, Ni, etc., and the excessive intake of heavy metals can cause numerous hazards to human bodies. For example, human exposure to Cu and Zn can cause some diseases, including capillary, liver, and kidney damages, as well as central nervous problems [10,11,12]. Cr can cause human skin congestion, erosion, ulcers, etc.; meanwhile, Cr can accumulate in livers, kidneys, and lungs, causing serious health hazards, such as liver and nerve damages, bronchitis, kidney and skin cancer, and diarrhea [13,14]. Pb can affect human nerves, interfere with the bio-synthesis of hemoglobin, cause anemia, motor and sensory abnormalities, lead to neurological disorders, hypertension, kidney disease, and anemia, and even cause death [15,16,17]. Therefore, the scientific, efficient, and complete removal of harmful heavy metal ions from water is essential [18].

Common methods for removing heavy metal ions from wastewater include redox, flocculation, bioremediation, ion exchange, adsorption, etc. [19,20]. Among these treatment methods, adsorption is widely used in the treatment of heavy metal ions in wastewater because of its high efficiency, low cost, and simple operation [21]. Among numerous adsorbents, chitosan is hydrophilic, biodegradable, and biocompatible [6,22]. Meanwhile, chitosan is a cheap, easily available, and environmentally friendly polymer material with abundant -NH_2_ and -OH on its surface, which can chelate with heavy metal ions such as Ag^+^, Cd^2+^, Cu^2+^, Fe^2+^, Pb^2+^, Zn^2+^, and Fe^3+^ in water to form stable chelates. Thus, chitosan is often used as an adsorbent to treat heavy metal ions in water bodies [23,24]. However, after chitosan adsorbs heavy metal ions, it is difficult to separate them from aqueous solution, which will bring secondary contamination [25,26,27]; therefore, the improvement of the separation effect is important when chitosan is applied to remove heavy metal ions by adsorption from wastewater.

Novel, highly efficient, and reusable magnetic adsorbents with easy separation after adsorption have potential applications in the treatment of wastewater containing heavy metal ions [28]. Ahmadi et al. [29] compared the adsorption of Cd^2+^ onto pure chitosan, γ-Fe_3_O_4_, and γ-Fe_3_O_4_-modified chitosan in aqueous solution; the results showed that the adsorption capacity order of Cd^2+^ was γ-Fe_3_O_4_-modified chitosan > pure chitosan > γ-Fe_3_O_4_. Cui et al. [30] prepared magnetic chitosan microspheres and studied their adsorption properties for Cu^2+^ in aqueous solution; the results showed that the adsorbent had an excellent adsorption capacity for Cu^2+^. At a temperature of 25 °C and a pH of 6, the adsorption could reach the saturation state at 6 h for the Cu^2+^ solution of 250 mg/L, and the adsorption capacity did not change much after the adsorbent was recycled five times. Fu et al. [31] also prepared magnetic chitosan microspheres with Fe_3_O_4_ as the magnetic core and studied the adsorption of Cu^2+^ (300 mg/L, 30 °C and pH = 5.0); the results showed that the adsorption capacity reached 32.89 mg/g. Chang et al. [32] prepared magnetic chitosan/graphene oxide adsorbent using the coprecipitation method. It was found that the adsorbent could adsorb Pb^2+^ (50 mg/L, room temperature and pH = 5), and the adsorption capacity reached 60.99 mg/g. Under the external magnetic field, the adsorbent displayed a good magnetic separation performance. After the adsorption material was recycled six times, the adsorption capacity only decreased by 16.72%, and it had an excellent recycling performance. Feng et al. [33] prepared composite magnetic chitosan materials for the adsorption of Cr^6+^ in aqueous solution. The results showed that when the temperature was 15 °C and the pH was 3.0, the adsorption capacity of Cr^6+^ was 47.8 mg/g, and the adsorption capacity could reach 79.1% after the adsorbent was reused four times.

As mentioned above, magnetic chitosan formed by compounding chitosan with magnetic ferric tetroxide has received much attention because of its homogeneous and regular porous structure, the large amount of -NH_2_ and -OH on its surface, its low cost, and good biocompatibility, as well as magnetic responsiveness, simple preparation conditions, and easy operation [34,35,36,37,38,39]. However, systematic studies on the adsorption removal and influencing adsorption factors of heavy metal ions onto magnetic nano-chitosan in aqueous solution, as well as the competitive adsorption among heavy metal ions, are not yet available. Therefore, on the basis on the adsorption kinetic and thermodynamic studies of single Cr^6+^, Cu^2+^, Pb^2+^, and Zn^2+^ onto magnetic nano-chitosan in aqueous solution, the adsorption influence factors of Cr^6+^, Cu^2+^, Pb^2+^, and Zn^2+^ onto magnetic nano-chitosan, and the competitive adsorption among Cu^2+^, Pb^2+^, and Zn^2+^ were investigated, which may provide some technical supports for the engineering application of magnetic nano-chitosan and the removal of Cr^6+^, Cu^2+^, Pb^2+^, and Zn^2+^ from wastewater.

## 2. Materials and Methods

### 2.1. Reagents

Chitosan (deacetylation degree ≥ 95%) was purchased from Macklin Biochemical Technology Co., Ltd. (Shanghai, China). Other chemicals and reagents utilized in the experiments were of analytical grade. FeCl_3_·6H_2_O was purchased from Tianjin Fuchen Chemical Reagent Co., Ltd. (Tianjin, China). FeCl_2_·4H_2_O was purchased from Tianjin Damao Chemical Reagent Factory (Tianjin, China). NaOH was purchased from Tianjin Guangfu Science and Technology Development Co., Ltd. (Tianjin, China). K_2_Cr_2_O_7_ was purchased from Tianjin Bodi Chemical Co., Ltd. (Tianjin, China). CuCl_2_·2H_2_O and ZnCl_2_ were purchased from Tianjin Komio Chemical Reagent Co., Ltd. (Tianjin, China). Pb(NO_3_)_2_, NaHCO_3_, CH_3_COOH, KBr, and C_2_H_6_O were purchased from Sinopharm Chemical Reagent Co., Ltd. (Shanghai, China).

### 2.2. Preparation of Magnetic Nano-Chitosan

The preparation of magnetic nano-chitosan was conducted by referring to the method adopted by Zhou and Guo et al. [32,38]. The detailed processes are as follows:

Chitosan solution preparation: 2 mL of acetic acid was placed into a 100 mL pre-washed beaker and 98 mL of ultrapure water was added. After 1 g of chitosan was weighed and added into the prepared acetic acid solution, they were ultrasonically dispersed and dissolved for 60 min to obtain chitosan solution.

Preparation of magnetic nano-Fe_3_O_4_: Magnetic nano-Fe_3_O_4_ was prepared using the co-precipitation method [39]. Specifically, 2.4 g of FeCl_2_·4H_2_O and 6.5 g of FeCl_3_·6H_2_O were weighed and placed into a 250 mL pre-washed beaker, and 225 mL of ultrapure water was added. Then, they were ultrasonically dispersed for 10 min, poured into a 500 mL pre-washed three-necked flask, and 90 mL of NaOH solution of 1 mol/L was dropped into the three-necked flask under room temperature, N_2_ atmosphere, and mechanical stirring. After that, they were aged for 1 h at 60 °C, washed with distilled water to neutral, magnetically separated, dried in an oven at 60 °C, and ground to produce black magnetic nano-Fe_3_O_4_.

Preparation of magnetic nano-chitosan: 80 mL of the prepared chitosan solution and 400 mg of the prepared magnetic nano-Fe_3_O_4_ were mixed in a 500 mL three-neck flask. They were diluted to 150 mL with ultrapure water, sonicated for 30 min at room temperature, heated in a constant temperature water bath at 30 °C, and stirred for 2 h with an electric stirrer at a constant speed. Then, a NaHCO_3_ solution of 10% was added dropwise until the solution became neutral, accompanied by the generation of dark brown bubbles. Finally, they were separated by a magnet, washed with distilled water to neutral, dried in an oven at 60 °C, and ground to obtain magnetic nano-chitosan.

### 2.3. Characterization of Magnetic Nano-Chitosan

The morphology of the prepared magnetic nano-Fe_3_O_4_ and magnetic nano-chitosan was observed using ultra-high-resolution transmission electron microscopy (TEM, JEM-2100, JEOL Electronics, Zhaodao, Japan). The physical phase structure of the two materials was determined using an X-Ray diffractometer (XRD, D8 Advance, Bruker, Ettlingen, Germany). The spectral characteristics of them were analyzed using a Fourier transform infrared spectrum (FT-IR, TENSOR27, Bruker, Ettlingen, Germany) in the range of 4000–300 cm^−1^ after they were pressed into a sample with KBr dried for more than 6 h at 100 °C. Their specific surface area and pore size were analyzed using a multi-station specific surface area and pore size analyzer (ASAP2460, Micromeritics, Atlanta, GA, USA). The specific surface area was calculated using the Brunauer–Emmet–Teller (BET) model. The pore size distribution was determined by the adsorption and desorption isotherm of N_2_ and was calculated using the Barrett–Joyner–Halenda (BJH) model.

### 2.4. Adsorption Experiments

The stock solutions of Cr^6+^, Cu^2+^, Pb^2+^, and Zn^2+^ at a concentration of 1 g/L were prepared with K_2_Cr_2_O_7_, CuCl_2_·2H_2_O, Pb(NO_3_)_2_, and ZnCl_2_, respectively, which were diluted into corresponding working solutions.

#### 2.4.1. Adsorption Dynamic Experiments

Nine pre-washed 20 mL headspace vials were prepared. In total, 50 mg of the prepared magnetic nano-chitosan and 10 mL of the prepared solution of Cr^6+^ at a concentration of 50 mg/L with a natural pH of 5 were added accurately into each headspace vial. They were shaken in a constant temperature water bath shaker of 298 K at 180 r/min; a headspace vial was taken out at 5, 10, 15, 20, 30, 45, 60, 120, and 180 min, respectively, they were separated by a magnet, and each sample was repeated three times. Meanwhile, the solutions of Cu^2+^, Pb^2+^, and Zn^2+^ at concentrations of 30, 50, and 20 mg/L, respectively, were prepared with a natural pH of 5, 6, and 4, respectively, for the adsorption kinetic experiments of them. The concentrations of Cr^6+^, Cu^2+^, Pb^2+^, and Zn^2+^ in the solution were analyzed using inductively coupled plasma atomic emission spectrometry (ICP-AES, Arcos, Spectro, Kleve, Germany).

#### 2.4.2. Adsorption Thermodynamics Experiments

The solutions (pH = 5) of Cr^6+^ with concentration gradients of 5, 10, 20, 50, 60, and 80 mg/L were prepared. Six pre-washed 20 mL headspace vials were prepared. In total, 50 mg of the prepared magnetic nano-chitosan was added in each headspace vial, and 10 mL of the prepared gradient solution of Cr^6+^ was added in turn. They were shaken for 120 min at 180 r/min in a constant temperature water bath shaker of 298 K, and were then magnetically separated by a magnet. Each sample was repeated three times. Meanwhile, the adsorption thermodynamic experiment was performed at 308 K and 318 K. In addition, the gradient solutions of 5, 10, 20, 50, 60, and 80 mg/L for Cu^2+^, 10, 20, 40, 50, 60, and 80 mg/L for Pb^2+^, and 5, 10, 20, 50, 60, and 80 mg/L for Zn^2+^ were prepared, with the natural pH of 5, 6, and 4 for Cu^2+^, Pb^2+^, and Zn^2+^, respectively. Under the temperature conditions of 298 K, 308 K, and 318K, respectively, the adsorption thermodynamics experiment was carried out in a constant temperature water bath oscillator for 120 min, with an oscillation frequency of 180 r/min and magnetic separation, and each sample was repeated three times. The concentrations of heavy metal ions in the solution were analyzed using ICP-AES.

#### 2.4.3. Influencing Factor Experiments

Initial concentrations of metal ions and temperatures: the experimental process was the same as that used for the adsorption thermodynamic experiments.

Magnetic nano-chitosan doses: Nine pre-washed 20 mL headspace vials were first dosed with 10, 20, 30, 40, 50, 60, 80, 100, and 120 mg of the prepared magnetic nano-chitosan, respectively, followed by 10 mL of solution (pH = 5) of Cr^6+^ at a concentration of 50 mg/L. Nine pre-washed 20 mL headspace vials were first added with 10, 20, 30, 50, 60, 80, 100, 120, and 140 mg of the prepared magnetic nano-chitosan, respectively, followed by 10 mL of solution (pH = 5) of Cu^2+^ at a concentration of 30 mg/L. Seven pre-washed 20 mL headspace vials were first dosed with 10, 20, 40, 60, 80, 120, and 150 mg of the prepared magnetic nano-chitosan, respectively, and then 10 mL of solution (pH = 6) of Pb^2+^ at a concentration of 50 mg/L was added. Eight pre-washed 20 mL headspace vials were first added with 10, 20, 30, 50, 60, 80, 100, and 120 mg of the prepared magnetic nano-chitosan, respectively, followed by 10 mL of solution (pH = 4) of Zn^2+^ at a concentration of 20 mg/L. They were shaken for 120 min at 180 r/min in a constant temperature water bath shaker of 298 K and were then separated by a magnet. Each sample was repeated three times. The concentrations of heavy metal ions were analyzed using ICP-AES.

Solution pH: 50 mg of the prepared magnetic nano-chitosan was added to 7 prepared 20 mL headspace vials, respectively, and 10 mL of solution of Cr^6+^ at a concentration of 50 mg/L with solution pH values of 1, 2, 3, 4, 5, 6, and 7 was added in turn. They were shaken for 120 min at 180 r/min in a constant temperature water bath shaker of 298 K, and then magnetically separated. Each sample was repeated three times. Experiments on the effect of pH on the adsorption of Cu^2+^, Pb^2+^, and Zn^2+^ were also carried out. The concentrations of Cu^2+^, Pb^2+^, and Zn^2+^ in the solution were 30, 50, and 20 mg/L, respectively, with solution pH values of 1, 2, 3, 4, 5, 6, and 7, respectively. The concentrations of heavy metal ions in the solution were analyzed using ICP-AES.

#### 2.4.4. Competitive Adsorption Experiments

Since Pb^2+^ and Cr_2_O_7_^2−^ can form PbCrO_4_ precipitate, the competitive adsorption of Cu^2+^, Pb^2+^, and Zn^2+^ onto the magnetic nano-chitosan in aqueous solution was mainly investigated. The competitive adsorption systems were designed as follows:

Single ion system: the concentration gradients of Cu^2+^, Pb^2+^, and Zn^2+^ were 10, 20, 40, 60, and 80 mg/L, 10, 20, 40, 60, and 80 mg/L, and 5, 10, 20, 60, and 80 mg/L, respectively.

Binary ion system: the concentrations of Zn^2+^ and Pb^2+^ were kept at 20 and 50 mg/L, respectively, and the concentrations of Cu^2+^ were set as 10, 20, 40, 60, and 80 mg/L, respectively, which obtains Cu^2+^- Zn^2+^ and Cu^2+^- Pb^2+^ binary systems, respectively. The concentrations of Cu^2+^ and Zn^2+^ were kept at 30 and 20 mg/L, respectively, and the concentrations of Pb^2+^ were designed as 10, 20, 40, 60, and 80 mg/L, respectively, obtaining Pb^2+^- Cu^2+^ and Pb^2+^- Zn^2+^ binary systems, respectively. The concentrations of Cu^2+^ and Pb^2+^ were kept at 30 and 50 mg/L, respectively, and the concentrations of Zn^2+^ were designed as 5, 10, 20, 60, and 80 mg/L, respectively, obtaining Zn^2+^- Cu^2+^ and Zn^2+^- Pb^2+^ binary systems, respectively.

Ternary ion system: the concentration gradients of Cu^2+^, Pb^2+^, and Zn^2+^ were all set as 5, 10, 20, 60, and 80 mg/L.

The competitive adsorption experiments were performed by adding 50 mg of the prepared magnetic nano-chitosan into each headspace vial, followed by adding 10 mL of the above concentration gradient solution of single or mixed ion solution in turn. They were shaken for 120 min at 180 r/min in a constant temperature water bath shaker of 298 K to reach adsorption equilibrium and were then separated using a magnet. Each sample was repeated three times. The concentrations of heavy metal ions in the solution were analyzed by ICP-AES.

### 2.5. Data Analysis

The adsorption capacity at the time t (q_t_, µmol/g), equilibrium adsorption capacity (q_e_, µmol/g), and removal efficiency (η, %) of the heavy metal ions at time t or equilibrium were calculated as follows.

(1) $$q_{t}=\frac{(c_{0}-c_{t})\times V}{M\times m}$$

(2) $$q_{e}=\frac{(c_{0}-c_{e})\times V}{M\times m}$$

(3) $$\eta =\frac{c_{0}-c_{t}/c_{e}}{c_{0}}\times 100\%$$

where c_0_, c_t_, and c_e_ (mg/L) are the initial, time t, and equilibrium concentrations of heavy metal ions, respectively; V (mL) is the volume of the solution; m (mg) is the weight of the adsorbent; and M (g/mol) is the molar mass of heavy metal ions.

Adsorption kinetic models, such as the pseudo-first-order kinetic model (4), pseudo-second-order kinetic model (5), Elovich model (6), and intraparticle diffusion model (7), were used to fit the adsorption kinetic curves [10,40,41,42,43].

(4) $$q_{t}=q_{e}(1-e^{-K_{1}t})$$

(5) $$q_{t}=\frac{K_{2}q_{e}{}^{2}t}{1+K_{2}q_{e}t}$$

(6) $$q_{t}=\alpha +K\times \ln t$$

(7) $$q_{t}=K_{d}t^{0.5}+C$$

where t (min) denotes the adsorption time; K_1_ (g·µmol^−1^·min^−1^) and K_2_ (g·µmol^−1^·min^−1^) are the rate constants for the pseudo-first-order and pseudo-second-order kinetic model, respectively; in the Elovich model, K (g·µmol^−1^·min^−1^) is the adsorption rate constant and a (g·µmol^−1^·min^−1^) is a constant; and K_d_ (µmol·g^−1^·min^−0.5^) is the intraparticle diffusion model rate constant and C is a constant term used to estimate the boundary layer thickness.

The Langmuir isotherm adsorption model describes an ideal single-molecule adsorption [43], commonly used in the adsorption of contaminants in liquid solutions, and the model is given in Equation (8); the R_L_ calculated in Equation (9) represents the affinity between the absorbents and adsorbates, and the adsorption is irreversible for R_L_ = 0, favorable for 0 < R_L_ < 1, linear for R_L_ = 1, and unfavorable for R_L_ > 1 [44]. The Freundlich isothermal adsorption model can be applied to a multilayer adsorption with the affinity on non-homogeneous surfaces [45], the heat of the adsorption decreases with an increasing surface coverage due to the inhomogeneity of the solid surface, and the proposed empirical model is given in Equation (10). The Temkin isothermal adsorption model assumes a linear decrease in adsorption heat at all surface locations due to adsorbent and adsorbate interactions [42,46], and the model is given in Equation (11).

(8) $$q_{e}=\frac{q_{m}K_{L}c_{e}}{1+K_{L}c_{e}}$$

(9) $$R_{L}=\frac{1}{\left(1+K_{L}c_{0}\right)}$$

(10) $$q_{e}=K_{F}c_{e}^{1/n}$$

(11) $$q_{e}=A\times \ln(K_{T}\times c_{e})$$

where q_m_ is the maximum adsorption capacity, µmol/g; K_L_ is the Langmuir model constant, L/µmol, and the surface adsorption capacity of the adsorbent is generally stronger when K_L_ is larger; K_F_ is the Freundlich model constant, (µmol/g)/(µmol/L)^1/n^; n is the index related to the adsorption strength, and 1/n < 1 indicates normal Freundlich adsorption, 0.1 < 1/n < 0.5 implies that there is an attraction between adsorbents and adsorbates that promotes adsorption, 1/n = 1 illustrates a linear adsorption generally occurring in relatively dilute solutions and on relatively low surface coverage adsorbents, and 1/n > 1 suggests that there is a synergistic adsorption and weak attraction between adsorbents and adsorbents, especially difficult adsorption at 1/n > 2; and A (J/mol) and K_T_ (L/µmol) are the Temkin model constants related to adsorption heat and binding energy, respectively.

The adsorption thermodynamics may reflect that the adsorption process is endothermic or exothermic, and temperature is an important factor affecting the adsorption. Thus, the adsorption thermodynamic parameters, i.e., Gibbs free energy (∆G, kJ/mol), the enthalpy of adsorption (∆H, kJ/mol), and the entropy of adsorption (∆S, J/(mol·K)), were analyzed and calculated as follows [45]:

(12) $$K_{D}=\frac{V_{s}q_{e}}{V_{e}c_{e}}$$

(13) $$\Delta G=-RT\ln K_{D}$$

(14) $$\ln K_{D}=\frac{\Delta S}{R}-\frac{\Delta H}{RT}$$

where K_D_ is the solid–liquid partition coefficient; V_s_ and V_e_ are the activity coefficients, both taken as 1; T is the absolute temperature, K; and R is the gas constant, 8.314 J/(mol∙K). When ∆G < 0, the reaction can proceed spontaneously; when ∆G = 0, the reaction is in equilibrium; and when ∆G > 0, the reaction cannot proceed spontaneously. ∆H > 0 indicates that the reaction is endothermic; ∆H < 0 implies that the reaction is exothermic. The actual reaction is always in the direction of increasing entropy, i.e., ∆S > 0. According to the principle of entropy increase, ∆S = 0 suggests that the reaction has reached equilibrium.

## 3. Results and Discussion

### 3.1. Characterization Results

**Figure 1:**
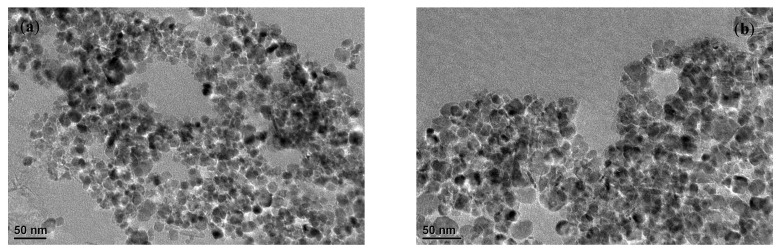

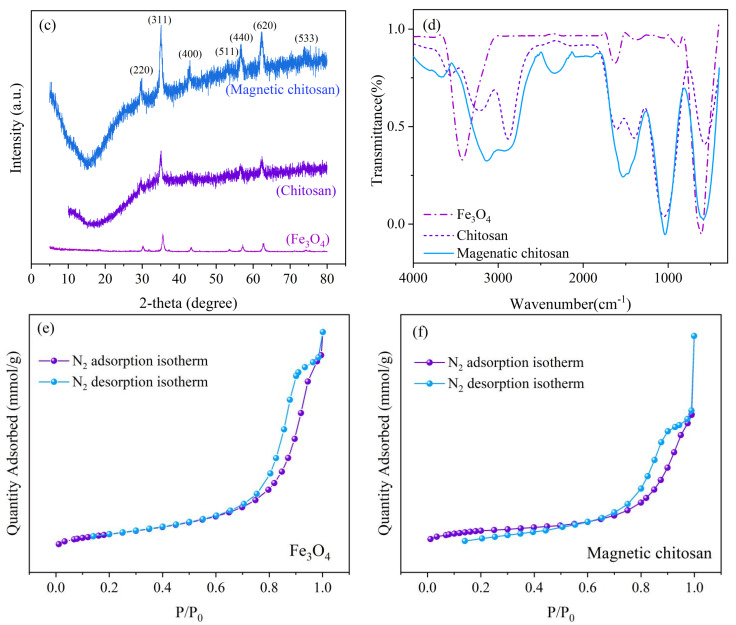
Images of TEM (**a**) magnetic nano-Fe_3_O_4_, (**b**) magnetic nano-chitosan), FT-IR (**c**), XRD (**d**), and N_2_ adsorption/desorption isotherms (**e**,**f**) of magnetic nano-Fe_3_O_4_ and magnetic nano-chitosan.

Figure 1 shows the characterization results of the prepared magnetic nano-Fe_3_O_4_ and magnetic nano-chitosan. From the TEM results in Figure 1a,b, the size of the prepared magnetic nano-Fe_3_O_4_ and magnetic nano-chitosan were below 50 nm and spherically arranged in an orderly manner, while the surface was not very smooth and there existed agglomerates. As shown in Figure 1c, the prepared magnetic nano-chitosan had seven characteristic peaks; the 2θ angles of the seven peaks were 30.1°, 35.5°, 43.1°, 57.1°, 62.5°, 71.5°, and 74.5°, respectively, with the corresponding crystallographic planes of (220), (311), (400), (511), (440), (620), and (533), respectively, which were the same as the crystallographic planes of the prepared magnetic nano-Fe_3_O_4_. There were the same diffraction peaks between the prepared magnetic nano-Fe_3_O_4_ and magnetic nano-chitosan, and no new diffraction peaks appeared in the prepared magnetic nano-chitosan, indicating that the synthesis of the magnetic nano-chitosan did not affect the crystal structure of Fe_3_O_4_ and did not change the crystallographic phase of Fe_3_O_4_. As shown in Figure 1d, the prepared magnetic nano-Fe_3_O_4_ had characteristic absorption peaks belonging to the stretching vibration of Fe-O at 560–600 cm^−1^ [35,37], indicating that the magnetic nano-Fe_3_O_4_ was successfully prepared. The main characteristic adsorption peaks of the prepared magnetic nano-chitosan were around 3433 cm^−1^ (O-H and N-H stretching vibration peaks), 2875 cm^−1^ (the stretching vibration peak of -CH), 1601 cm^−1^ (the bending vibration peak of -NH in -NH_2_), and 560 cm^−1^ (Fe-O stretching vibration peak) [35,46,47], indicating that chitosan was successfully loaded onto the magnetic nano-Fe_3_O_4_. As shown in Figure 1e,f, the prepared magnetic nano-Fe_3_O_4_ and magnetic nano-chitosan exhibited typical type IV isotherm adsorption characteristics, indicating that there was a relatively strong interaction of nitrogen onto the sample surfaces, the prepared magnetic nano-Fe_3_O_4_ was a mesoporous material (pore width: 2–50 nm), and the magnetic nano-chitosan was a microporous material (pore width: 0–2 nm). The specific surface area of the prepared magnetic nano-Fe_3_O_4_ based on the BET model was 17.45 m^2^/g; without a microporous surface area, the total pore volume was 0.14 cm^3^/g, and the average adsorption pore width measured by the BJH model was 7.84 nm. The prepared magnetic nano-chitosan had a specific surface area of 1.13 m^2^/g, no mesoporous surface area, a total pore volume of 0.02 cm^3^/g, and a mean adsorption pore width of 9.15 nm. Compared with that of the prepared magnetic nano-Fe_3_O_4_, the specific surface area of the prepared magnetic nano-chitosan reduced, while the pore width became larger.

### 3.2. Adsorption Kinetics

**Figure 2:**
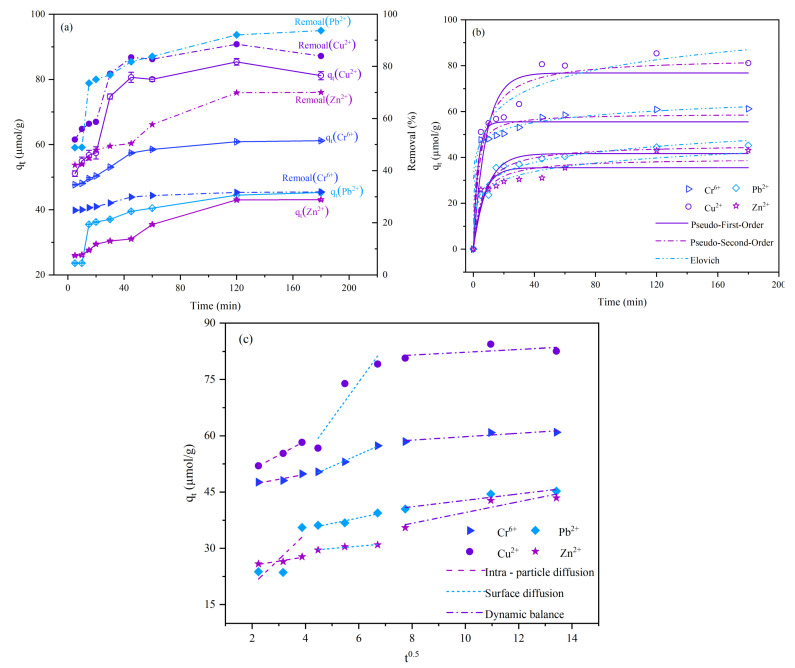
(**a**) Adsorption kinetics of Cr^6+^, Cu^2+^, Pb^2+^, and Zn^2+^ onto magnetic nano-chitosan in aqueous solution; (**b**) fitting by pseudo-first-order, pseudo-second-order, and Elovich kinetic models; and (**c**) fitting of intraparticle diffusion kinetic model.

Figure 2a depicts the adsorption kinetics curves of Cr^6+^, Cu^2+^, Pb^2+^, and Zn^2+^ onto magnetic nano-chitosan in aqueous solution, respectively. The adsorption process was divided into two stages, i.e., the adsorption capacity and removal rate of Cr^6+^ as well as Cu^2+^, Pb^2+^, and Zn^2+^ increased sharply in the first 60 and 120 min, respectively (fast adsorption stage); the adsorption capacity and removal rate changed slowly (slow adsorption stage). In general, when the adsorption involves a surface reaction process, the initial adsorption is relatively rapid due to the large number of available adsorption sites on the adsorbent; then, as the number of available adsorption sites gradually decreases, the adsorption slows down and reaches an equilibrium [47,48,49]. In addition, the experimentally obtained adsorption capacity (q_exp_) presented the order of Cu^2+^ (81.141 μmol/g) > Cr^6+^ (61.208 μmol/g) > Pb^2+^ (45.276 μmol/g) > Zn^2+^ (43.092 μmol/g), and the maximum removal rate (η) followed the order of Pb^2+^ (93.72%) > Cu^2+^ (88.48%) > Zn^2+^ (70.03%) > Cr^6+^ (31.83%).

Figure 2b shows that the adsorption kinetic curves of Cr^6+^, Cu^2+^, Pb^2+^, and Zn^2+^ onto magnetic nano-chitosan in aqueous solution were fitted by using pseudo-first-order, pseudo-second-order, and Elovich kinetic models. As shown in Figure 2b, the adsorption of metal ions onto magnetic nano-chitosan could reach the equilibrium of adsorption at 30 min for Cr^6+^ and at 60 min for Cu^2+^, Pb^2+^, and Zn^2+^. Table 1 shows the fitted results of the parameters of pseudo-first-order, pseudo-second-order, and Elovich kinetic models for the adsorption kinetics of Cr^6+^, Cu^2+^, Pb^2+^, and Zn^2+^ in aqueous solution by magnetic nano-chitosan. As shown in Table 1, the fitted correlation coefficient (R^2^) by the pseudo-second-order kinetic model for Cr^6+^, Cu^2+^, Pb^2+^, and Zn^2+^ was larger than that by the pseudo-first-order kinetic model, with R^2^ being greater than 0.960, and the equilibrium adsorption capacity (q_e_) fitted by the pseudo-second-order kinetic model was also closer to the q_exp_, indicating that the pseudo-second-order kinetic model could well describe the adsorption kinetic process, including liquid film diffusion, surface adsorption, internal diffusion, and chemical bound formation. It can be inferred that the adsorption of Cr^6+^, Cu^2+^, Pb^2+^, and Zn^2+^ in aqueous solution by magnetic nano-chitosan was dominated by chemisorption. The Elovich kinetic model also provided good fits to the adsorption kinetic curves, with R^2^ > 0.96. The Elovich kinetic model is mainly used to study the non-homogeneous diffusion process in the combined presence of adsorbent adsorption behavior and adsorbate diffusion, which does not predict any conventional mechanism [50,51] and is suitable for the reaction process with large activation energies. The good fitting indicates that the adsorption process is a non-homogeneous diffusion process regulated by a combination of the reaction rate and diffusion factors. 

Figure 2c shows the fitting results of the intraparticle diffusion model. As shown in Figure 2c, the adsorption of Cr^6+^, Cu^2+^, Pb^2+^, and Zn^2+^ onto magnetic nano-chitosan in aqueous solution was a combined existence process of adsorption and diffusion rather than a simple first-order reaction; the whole adsorption process was divided into the dynamic processes of fast surface adsorption, intraparticle diffusion, and adsorption and desorption equilibrium, in which the equilibrium dynamic process of intraparticle diffusion was relatively fast and cannot be regarded as the rate-limiting step. Table 2 shows the fitted parameter values of the intraparticle diffusion model for Cr^6+^, Cu^2+^, Pb^2+^, and Zn^2+^ adsorption in aqueous solutions by magnetic nano-chitosan. From Table 2, Cr^6+^ and Cu^2+^ followed K_2d_ > K_1d_ > K_3d_, indicating the intraparticle diffusion rate > surface diffusion rate > equilibrium dynamic rate; Pb^2+^ presented K_1d_ > K_2d_ > K_3d_, showing the surface diffusion rate > intraparticle diffusion rate > the equilibrium dynamic rate; and Zn^2+^ exhibited K_3d_ > K_1d_ > K_2d_, indicating the equilibrium dynamic rate > surface diffusion rate > intraparticle diffusion rate. Meanwhile, the fitted curves of the intraparticle diffusion model did not pass through the origin, q_t_ and t^0.5^ were nonlinear relations, and the C value was not zero, indicating that other mechanisms besides intraparticle diffusion might have been involved. The adsorption process was possibly controlled by the boundary layer [52]. The reason is that the boundary layer effect is stronger when the C value (intercept value) is larger [52,53].

### 3.3. Adsorption Thermodynamics

**Figure 3:**
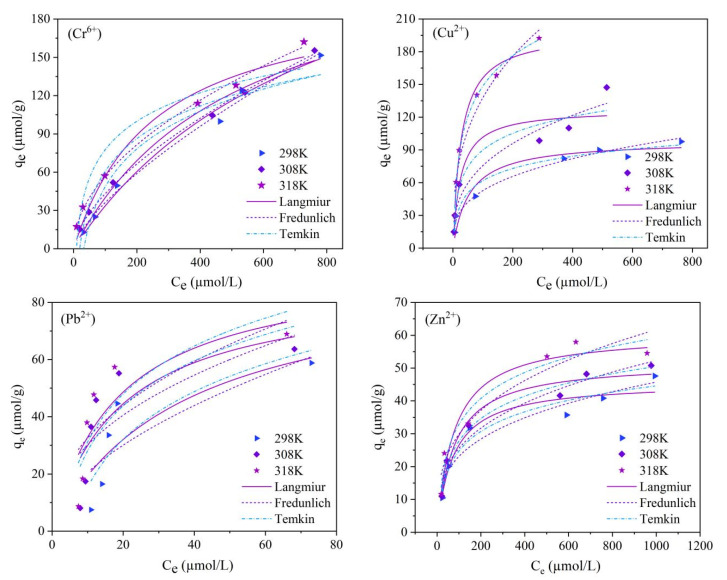
Fitting of Langmuir, Freundlich, and Temkin isotherm models to the adsorption of Cr^6+^, Cu^2+^, Pb^2+^, and Zn^2+^ onto magnetic nano-chitosan in aqueous solution.

Adsorption isotherms show how adsorbent molecules are distributed between liquid and solid two phases when the adsorption process reaches an equilibrium [54,55], and they help to determine the properties of the adsorbent, such as the pore size, pore volume, and surface area [23]. In this study, the isothermal adsorption models of Langmuir, Freundlich, and Temkin were used to fit the adsorption thermodynamic curves of Cr^6+^, Cu^2+^, Pb^2+^, and Zn^2+^ onto magnetic nano-chitosan in aqueous solution at 298 K, 308 K, and 318 K, respectively, and the results are presented in Figure 3 and Table 3. As shown in Figure 3, the q_e_ of Cr^6+^, Cu^2+^, Pb^2+^, and Zn^2+^ increased exponentially with the increase in the equilibrium concentration (c_e_) of heavy metal ions in the solution, and the q_e_ of Cr^6+^, Cu^2+^, Pb^2+^, and Zn^2+^ increased rapidly at the c_e_ of less than 200, 100, 20, and 200 µmol/L, respectively. Among them, temperature had the strongest effect on Cu^2+^ adsorption, followed by Pb^2+^ and Zn^2+^ adsorption, and was relatively weak for the Cr^6+^ adsorption influence. At the same c_e_, the higher the temperature, the greater the q_e_ of Cr^6+^, Cu^2+^, Pb^2+^, and Zn^2+^, indicating that the adsorption process was endothermic and the increase in the temperature was beneficial to the adsorption reaction.

As shown in Table 3, the R^2^ fitted by the Langmuir isothermal adsorption model for the adsorption thermodynamics of Cr^6+^, Cu^2+^, Pb^2+^, and Zn^2+^ onto magnetic nano-chitosan in the solution was slightly larger than that fitted by Freundlich and Temkin isothermal adsorption models, and the maximum adsorption capacity (q_m_) obtained by the Langmuir isothermal adsorption model for Cu^2+^ and Zn^2+^ was very close to that of the q_exp_, indicating that the Langmuir isothermal adsorption model could well describe the adsorption thermodynamics and that the adsorption belonged to monolayer adsorption. Among them, the R_L_ values of Cr^6+^, Cu^2+^, Pb^2+^, and Zn^2+^ were in the range of 0 to 1 at different temperatures and concentrations, indicating that the affinity between magnetic nano-chitosan and heavy metal ions was favorable for adsorption. The q_m_ of magnetic nano-chitosan for Cr^6+^, Cu^2+^, Pb^2+^, and Zn_2+_ at 318 K obtained by fitting the Langmuir adsorption isotherm model were up to 301.057, 198.861, 121.942, and 62.727 µmol/g, respectively. Meanwhile, the q_m_ obtained by fitting the Langmuir adsorption isotherm model shows that the adsorption effect of magnetic nano-chitosan for Cr^6+^ in aqueous solution was more obvious, followed by Pb^2+^ and Cu^2+^, and the adsorption effect of Zn^2+^ was relatively low. In addition, the values of 1/n by fitting the Freundlich adsorption isotherm model were all less than 1, indicating the existence of an attraction between the adsorbent surface and adsorbate that promotes adsorption. The fitted constant A of the Temkin isotherm model suggests that the heat of adsorption increased with the increase in temperature, further indicating that the adsorption process was endothermic.

The adsorption thermodynamic parameters, including ∆G, ∆H, and ∆S, are shown in Table 4. As shown in Table 4, the values of ∆G were all below 0 and decreased with the increase in temperature, indicating that the adsorption processes of Cr^6+^, Cu^2+^, Pb^2+^, and Zn^2+^ onto magnetic nano-chitosan in aqueous solution were spontaneous. The values of ∆H were all above 0, further indicating that the adsorption processes were endothermic and that the increase in temperature is favorable for adsorption.

### 3.4. Factors Influencing Adsorption

#### 3.4.1. Adsorbent Dosage and Solution pH

**Figure 4:**
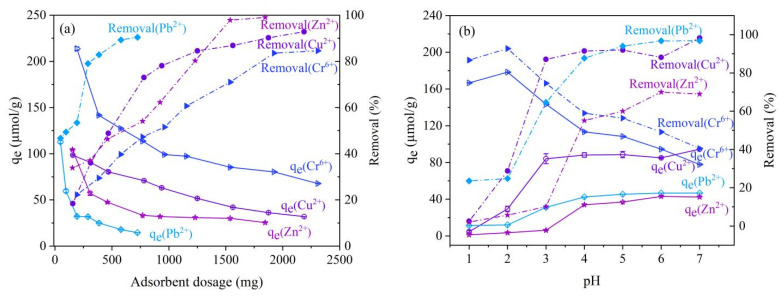
Effects of absorbent dosages (**a**) and solution pH (**b**) on the adsorption of Cr^6+^, Cu^2+^, Pb^2+^, and Zn^2+^ onto magnetic nano-chitosan in aqueous solution.

Figure 4 shows the effects of the magnetic nano-chitosan dosage and solution pH on the adsorption and removal of Cr^6+^, Cu^2+^, Pb^2+^, and Zn^2+^ in aqueous solution. As shown in Figure 4a, the η of Cr^6+^, Cu^2+^, Pb^2+^, and Zn^2+^ first showed a linear and rapid increase, followed by a slow increase towards equilibrium; the q_e_ decreased from fast to slow with the dosage increase of magnetic nano-chitosan. When the concentrations of Cr^6+^, Cu^2+^, Pb^2+^, and Zn^2+^ in aqueous solution are constant, increasing the dosage of magnetic nano-chitosan means increasing the active adsorption sites where Cr^6+^, Cu^2+^, Pb^2+^, and Zn^2+^ can be adsorbed, so the adsorption removal efficiency gradually increases with the increase in the magnetic nano-chitosan dose; when the amount of magnetic nano-chitosan dosed is too high, the number of adsorption sites is much larger than the amount of Cr^6+^, Cu^2+^, Pb^2+^, and Zn^2+^, then the adsorption capacity per unit adsorbent reduces instead, i.e., the adsorbent utilization rate reduces. As shown in Figure 4b, the q_e_ and η of Cr^6+^ first increased and then decreased with the increase in the solution’s pH; the q_e_ and η decreased from 92.758 to 40.498 µmol/g and from 95% to 40% at the solution’s pH of 2–7. The q_e_ and η of Cu^2+^, Pb^2+^, and Zn^2+^ increased as the solution’s pH increased, while it basically ceased to change when the solution’s pH was above four; the maximum η could reach 100% for Cu^2+^ and Pb^2+^ and 75% for Zn^2+^. When the pH of the solution is low, the concentration of H^+^ in the solution is high, and the amino groups on the surface of magnetic nano-chitosan are prone to a protonation reaction to form -NH_3_^+^, which can decrease the number of amino groups that produce the effective complexation of Cu^2+^, Pb^2+^, and Zn^2+^. Therefore, the adsorbent has a low adsorption capacity for Cu^2+^, Pb^2+^, and Zn^2+^ under a low pH condition and the q_e_ are also relatively small. As the pH of the solution increases, the concentration of H^+^ in the solution gradually decreases, the competition ability between H^+^ and Cu^2+^, Pb^2+^, and Zn^2+^ gradually decreases, the adsorption sites on the surface of magnetic nano-chitosan are released, and the adsorbent protonation effect is gradually weakened and the electrostatic repulsion is also reduced. Under this condition, the magnetic nano-chitosan adsorption capacities for Cu^2+^, Pb^2+^, and Zn^2+^ gradually increased, leading to an increase in the adsorption capacity of Cu^2+^, Pb^2+^, and Zn^2+^ [56]. At the solution of pH < 2, Cr^6+^ mainly exists as HCrO_4_^−^ , and the magnetic nano-chitosan surface is positively charged, HCrO_4_^−^ will be adsorbed on the magnetic nano-chitosan, thus the q_e_ of Cr^6+^ increases; when the solution’s pH increases from two, the concentration of OH^−^ increases, and OH^−^ will compete with CrO_4_^2−^, so the q_e_ of Cr^6+^ decreases [57].

#### 3.4.2. Initial Concentration of Heavy Metal Ions and Temperature

**Figure 5:**
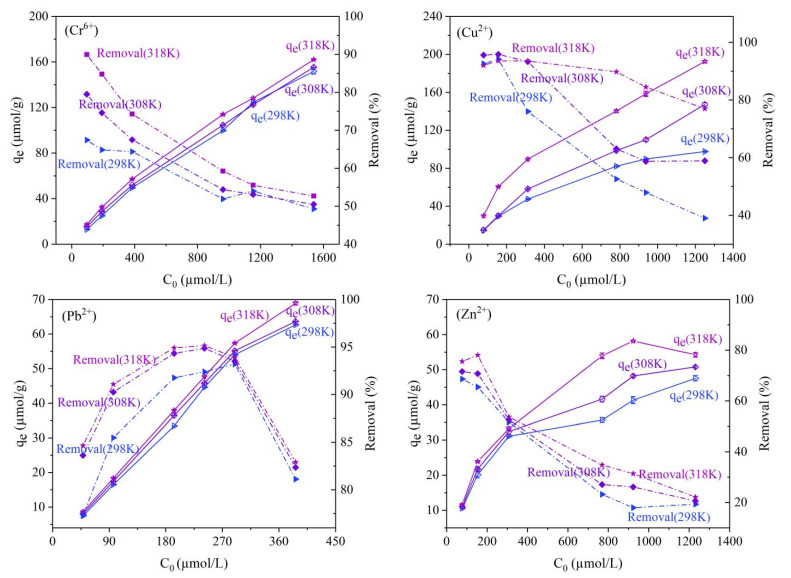
Effects of initial concentration and temperature on adsorption of Cr^6+^, Cu^2+^, Pb^2+^, and Zn^2+^ onto magnetic nano-chitosan in aqueous solution.

Figure 5 shows the effect of the initial concentration of metal ions and temperature on the adsorption and removal of Cr^6+^, Cu^2+^, Pb^2+^, and Zn^2+^ from aqueous solution by magnetic nano-chitosan. As can be seen from Figure 5, the q_e_ of Cr^6+^, Cu^2+^, Pb^2+^, and Zn^2+^ onto magnetic nano-chitosan showed an overall linear increase when the initial concentrations of metal ions increased, the temperature had a weak influence on the adsorption of Cr^6+^ and Pb^2+^ and a strong influence on Cu^2+^ and Zn^2+^ at the same initial concentration, and the higher the temperature, the greater the q_e_, i.e., 318 K > 308 K > 298 K; the η of Cr^6+^, Cu^2+^, and Zn^2+^ decreased continuously with the increase in the initial concentration of metal ions, and the lower the temperature, the lower the η at the same initial concentration, i.e., 318 K > 308 K > 298 K. With the increase in the initial concentration of metal ions, the η of Pb^2+^ increased first and then decreased, and reached a maximum of 95% when the initial concentration of Pb^2+^ increased to 289.855 µmol/L. As the initial concentration of Pb^2+^ continued to increase, the η of Pb^2+^ decreased rapidly. The probable reason for this is that the process of Pb^2+^ reaching adsorption equilibrium is relatively slow; the removal rate gradually increases with the increase in its initial concentration in the solution and has started to decrease when its adsorption sites reach saturation.

### 3.5. Competitive Adsorption of Metal Ions

**Figure 6:**
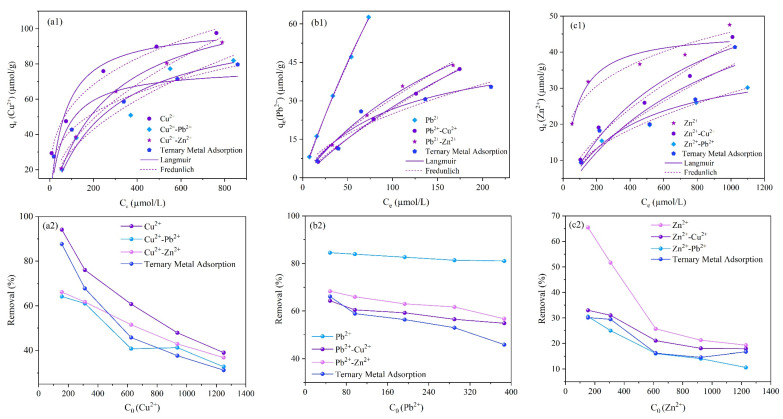
Fitting results of Langmuir and Freundlich isothermal adsorption models of Cu^2+^ (**a1**), Pb^2+^ (**b1**), and Zn^2+^ (**c1**) competitive adsorption experiment; removal rates of Cu^2+^ (**a2**), Pb^2+^ (**b2**), and Zn^2+^ (**c2**) competitive adsorption.

Figure 6a1–c1 shows the fitted results of the Langmuir and Freundlich isothermal adsorption models for the single and competitive adsorption of Cu^2+^, Pb^2+^, and Zn^2+^ onto magnetic nano-chitosan in aqueous solution as well as the comparison of the adsorption capacities and removal rates of Cu^2+^, Pb^2+^, and Zn^2+^ in single, binary, and ternary ion systems. The q_e_ of Cu^2+^, Pb^2+^, and Zn^2+^ in single, binary, and ternary ion systems showed an increasing trend with the increase in the metal ion equilibrium concentration. In binary and ternary iron systems, the q_e_ of Cu^2+^, Pb^2+^, and Zn^2+^ decreased compared to the corresponding single system. The experimentally obtained q_exp_ of Cu^2+^, Pb^2+^, and Zn^2+^ in the binary ion system of Cu^2+^-Pb^2+^ and Cu^2+^-Zn^2+^, Pb^2+^-Cu^2+^ and Pb^2+^-Zn^2+^, and Zn^2+^-Cu^2+^ and Zn^2+^-Pb^2+^ reduced by 16.01% and 5.44%, 32.28% and 29.97%, and 7.12% and 45.01%, respectively, indicating that the influence presented Pb^2+^ to Cu^2+^ >> Zn^2+^ to Cu^2+^, Cu^2+^ on Pb^2+^ > Zn^2+^ on Pb^2+^, and Pb^2+^ to Zn^2+^ >> Cu^2+^ to Zn^2+^. The q_e_ of Cu^2+^, Pb^2+^, and Zn^2+^ in the ternary ion system decreased by 18.34%, 43.36%, and 13.02%, respectively, suggesting that mutual effects among the metal ions existed.

The removal efficiency of magnetic nano-chitosan for Cu^2+^, Pb^2+^, and Zn^2+^ was also reduced in the presence of coexisting ions. As shown in Figure 6a2, the influence of Pb^2+^ to Cu^2+^ was stronger than that of Zn^2+^ to Cu^2+^. It can be seen from Figure 6b2 that the effect of coexisting ions on Pb^2+^ exhibited Cu^2+^ and Zn^2+^ > Cu^2+^ > Zn^2+^. From Figure 6c2, the influence of coexisting ions on Zn^2+^ presented Pb^2+^ stronger than Cu^2+^ and Pb^2+^ stronger than Cu^2+^. Overall, it seems that the mutual competitive adsorption of Cu^2+^, Pb^2+^, and Zn^2+^ was obvious, with Pb^2+^ being relatively strongly affected by the coexisting ions.

The decrease in the adsorption capacity of Cu^2+^, Pb^2+^, and Zn^2+^ in the binary and ternary ion systems compared to that in the single ion system was mainly due to the competitive adsorption effects of coexisting Pb^2+^ and Zn^2+^, Zn^2+^ and Cu^2+^, and Cu^2+^ and Pb^2+^. Table 5 shows the fitted results of the Langmuir and Freundlich isothermal adsorption models for the competitive adsorption of Cu^2+^, Pb^2+^, and Zn^2+^ onto magnetic nano-chitosan in aqueous solution. As shown in Table 5, the R^2^ fitted by the Langmuir isothermal adsorption model for the competitive adsorption of Pb^2+^-Cu^2+^, Pb^2+^-Zn^2+^, and Zn^2+^-Cu^2+^ in aqueous solution onto magnetic nano-chitosan was above 0.930, and the R^2^ fitted by the Freundlich isothermal adsorption model was above 0.940, indicating good fitting effects.

**Figure 7:**
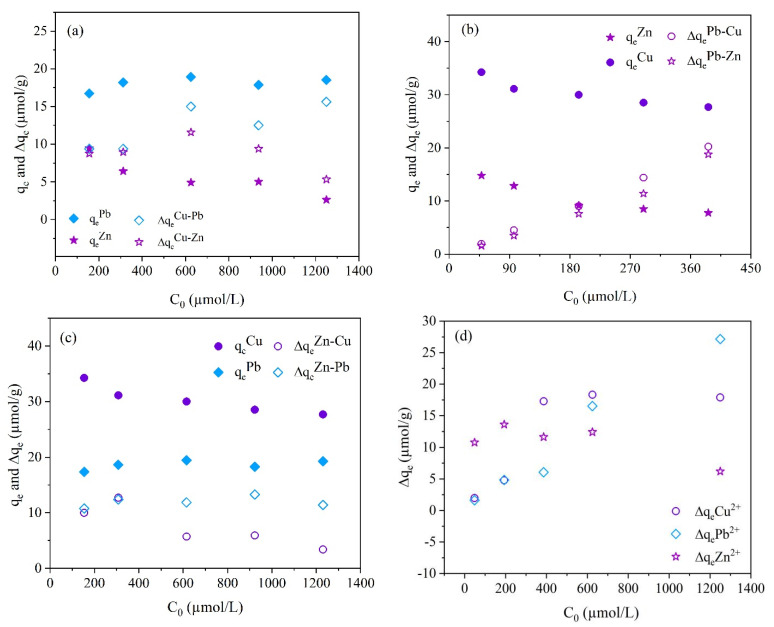
Competitive adsorption of Cu^2+^, Pb^2+^, and Zn^2+^ (∆q_e_^Cu-Pb^, ∆q_e_^Cu-Zn^, ∆q_e_^Pb-Cu^, ∆q_e_^Pb-Zn^, ∆q_e_^Zn-Cu^ and ∆q_e_^Zn-Pb^ represent the absolute equilibrium adsorption capacity of Cu^2+^ (**a**), Pb^2+^ (**b**), and Zn^2+^ (**c**) in binary ion system, respectively; ∆q_e_Cu^2+^, ∆q_e_Pb^2+^, and ∆q_e_Zn^2+^ represent the absolute equilibrium adsorption capacity of Cu^2+^, Pb^2+^, and Zn^2+^ in ternary ion system (**d**), respectively).

In order to clearly determine the specific effects of coexisting metal ions on the adsorption of Cu^2+^, Pb^2+^, and Zn^2+^, the absolute equilibrium adsorption capacity (Δq_e_ = q_e_–q_e_^competitor^) of the target ions was compared and the equilibrium adsorption capacity of the competitor (q_e_^competitor^ of Cu^2+^/Zn^2+^/Pb^2+^) was further conducted. From Figure 7a, when Pb^2+^ and Zn^2+^ were competitors, with the increase in the initial concentration of target Cu^2+^, the q_e_ of Pb^2+^ kept at a constant level, the ∆q_e_^Cu-Pb^ first increased and then remained at a constant level, indicating no influence between Cu^2+^ and Pb^2+^; the q_e_ of Zn and ∆q_e_^Cu-Zn^ exhibited a decreasing trend, illustrating the existing mutual inhibition effects between Cu^2+^ and Zn^2+^. From Figure 7b, when Cu^2+^ and Zn^2+^ were competitors, with the increase in the target Pb^2+^ initial concentration, the q_e_ of Cu^2+^ and Zn^2+^ decreased and the ∆q_e_^Pb-Cu^ and ∆q_e_^Pb-Zn^ increased, implying an inhibition of Pb^2+^ to Cu^2+^ and Zn^2+^. From Figure 7c, when Cu^2+^ and Pb^2+^ were competitors, with the increase in the target Zn^2+^ initial concentration, the q_e_ of Cu^2+^ and ∆q_e_^Zn-Cu^ decreased, showing a mutual inhibition between Zn^2+^ and Cu^2+^; the q_e_ of Pb^2+^ and ∆q_e_^Zn-Pb^ were kept a constant level, indicating no effect of Zn^2+^ on Pb^2+^ as well as the inhibition of Pb^2+^ to Zn^2+^. From Figure 7d, in the ternary ion system, with the increase in the metal ion initial concentration (C_0_), ∆q_e_Pb^2+^ increased linearly, ∆q_e_Cu^2+^ first increased and then tended to stable, and ∆q_e_Zn^2+^ first increased and then decreased. They presented ∆q_e_Zn^2+^ > ∆q_e_Cu^2+^ ≈ ∆q_e_Pb^2+^ at the C_0_ of < 200 µmol/L, ∆q_e_Cu^2+^ > ∆q_e_Zn^2+^ or ∆q_e_Pb^2+^ at the C_0_ of 200–600 µmol/L, and ∆q_e_Pb^2+^ > ∆q_e_Cu^2+^ > ∆q_e_Zn^2+^ at the C_0_ of > 600 µmol/L. These indicate that the inhibition of coexisting ions to Pb^2+^ adsorption gradually decreased with the increase in the metal ion initial concentration, and the inhibition of coexisting ions to Zn^2+^ and Cu^2+^ adsorption first decreased and then tended to be strong or stable, indicating that the three heavy metal ions have mutual effects when they coexist, and the competitive adsorption was obvious.

The number of metal ions adsorbed on the surface of magnetic chitosan is not only related to the characteristics of the adsorbent but is also related to other factors, such as the hydration radius, ion-exchange, metal ion complexation, and electrostatic interactions, which were the main governing mechanisms for almost all the chitosan-based materials and usually function together to achieve the adsorption of metal ions from the aqueous solution [58]. The present competitive adsorption results showed that the competitive adsorption order of the three metal ions was Cu^2+^ > Pb^2+^ > Zn^2+^. Generally, the metal adsorption affinity increases with the increasing hydrolysis constant of the metal ions. Previous studies have shown that the order of hydrolysis constants of the metal ions studied is Pb^2+^ (10^−7.71^) > Cu^2+^ (10^−8^) > Zn^2+^ (10^−9^). The electronegativity of metal ions studied followed Pb^2+^(2.33) > Cu^2+^ (1.96) > Zn^2+^(1.65), indicating that Pb^2+^ has a greater competitive advantage in adsorption [59,60]. Meanwhile, the hydration radius of Pb^2+^ (4.01 Å) is smaller than that of Cu^2+^ (4.19 Å) and Zn^2+^ (4.30 Å), which is consistent with the metal adsorption capacity of Pb^2+^ and Zn^2+^ [39,43]. A previous study also demonstrated that metal ions with smaller ionic diameters have higher adsorption rates [60,61]. The adsorption capacity of magnetic chitosan for Cu^2+^ was higher than that of Pb^2+^ and Zn^2+^, which can be attributed to the formation of Cu⋯NH-complex [62], in which a pair of lone electrons in the nitrogen atom are contributed to the common bond between N and Cu^2+^.

## 4. Conclusions

The adsorption kinetics of Cr^6+^, Cu^2+^, Pb^2+^, and Zn^2+^ onto magnetic mano-chitosan in aqueous solution was well described by the pseudo-second kinetic model, being mainly chemisorption. The adsorption thermodynamics was well fitted by the Langmuir isothermal adsorption model, the adsorption was mainly unimolecular layer adsorption, and the q_m_ of Cr^6+^, Cu^2+^, Pb^2+^, and Zn^2+^ at 318 K was 301.057, 198.861, 121.9421, and 62.727 µmol/g, respectively. With the dosage increase in magnetic nano-chitosan, the q_e_ of Cr^6+^, Cu^2+^, Pb^2+^, and Zn^2+^ decreased from fast to slowly and their η first increased and then slowly changed. With the increase in the solution’s pH, the q_e_ and η of Cr^6+^ first increased and then decreased, being up to their maximum values at pH = 2; the q_e_ and η of Cu^2+^, Pb^2+^, and Zn^2+^ increased at the solution pH of < 4, and slowly changed at the solution pH of > 4. With the increase in the initial concentration of metal ions, the q_e_ increased, the temperature was higher, and the q_e_ was larger, i.e., 318 K > 308 K > 298 K; the η of Cr^6+^, Cu^2+^, and Zn^2+^ decreased continuously, while the η of Pb^2+^ showed a trend of first increasing and then decreasing, and the adsorption of metal ions was a spontaneous and feasible endothermic process. The q_e_ and η in the binary and ternary ion systems decreased compared to those in the single ion system. There was the mutual adsorption influence among metal ions when they co-existed. In the ternary ion system, the q_m_ of Cu^2+^ could be up to 78.4616 μmol/g. The current study’s results provide theoretical support for the simultaneous treatment of harmful metal ions in wastewater by magnetic mano-chitosan.

In the present study, the single and completive adsorption of Cr^6+^, Cu^2+^, Pb^2+^, and Zn^2+^ onto magnetic mano-chitosan in aqueous solution were systematically studied; however, the adsorption capacities were relatively moderate. Therefore, the recycle of magnetic mano-chitosan was not conducted. In future studies, the magnetic nano-Fe_3_O_4_ will be first salinized or aminated, and then cross-linked with chitosan by using glutaraldehyde to improve the adsorption capacities of metal ions. Additionally, the recycle of the adsorbents will also be studied.

## Figures and Tables

**Table 1:** Fitted parameter values of pseudo-first-order, pseudo-second-order, and Elovich kinetic models.

Ions	q_exp_ (µmol/g)	Pseudo-First-Order			Pseudo-Second-Order			Elovich		
		K_1_ (g·µmol^−1^·min^−1^)	q_e_ (µmol/g)	R^2^	K_2_ (g·µmol^−1^·min^−1^)	q_e_ (µmol/g)	R^2^	α (g·µmol^−1^·min^−1^)	K (g·µmol^−1^·min^−1^)	R^2^
Cr^6+^	61.208	0.4185	57.283	0.9397	0.0119	60.626	0.9757	39.465	4.7523	0.9946
Cu^2+^	81.141	0.1492	81.260	0.8702	0.0023	81.575	0.9390	33.499	11.878	0.9622
Pb^2+^	45.276	0.1299	43.287	0.9451	0.0037	45.748	0.9722	15.360	6.559	0.9643
Zn^2+^	43.092	0.2043	37.951	0.8119	0.0071	42.126	0.9021	14.951	5.692	0.9695

**Table 2:** Fitted parameter values of intraparticle diffusion kinetic model.

Ions	Intra-Particle Diffusion								
	K_1d_ (µmol·g^−1^·min^−0.5^)	C_1_	R^2^	K_2d_ (µmol·g^−1^·min^−0.5^)	C_2_	R^2^	K_3d_ (µmol·g^−1^·min^−0.5^)	C_3_	R^2^
Cr^6+^	1.878	46.217	0.8470	3.371	37.629	0.9938	0.6537	57.386	0.8404
Cu^2+^	3.953	43.843	0.9986	13.474	36.014	0.8778	0.898	84.348	0.3308
Pb^2+^	11.653	21.726	0.6685	1.891	31.474	0.9310	1.118	37.141	0.9129
Zn^2+^	1.450	24.154	0.9300	0.758	27.656	0.9511	2.001	31.342	0.8667

**Table 3:** Fitting parameters of Langmuir, Freundlich, and Temkin isothermal models.

Ions	T (K)	q_exp_ (µmol/g)	Langmuir			Freundlich			Temkin		
			K_L_ (L/µmol)	q_m_ (µmol/g)	R^2^	1/n	K_F_ (µmol/g)/(µmol/L)^1/n^	R^2^	A (J/mol)	K_T_ (L/µmol)	R^2^
Cr^6+^	298	151.635	0.004	203.360	0.9915	0.74	1.619	0.8917	35.61	0.039	0.9462
	308	155.365	0.002	248.083	0.9982	0.64	2.751	0.9829	35.87	0.062	0.9340
	318	162.096	0.001	301.057	0.9977	0.52	5.840	0.9695	36.47	0.112	0.9318
Cu^2+^	298	97.580	0.027	98.617	0.9849	0.33	11.071	0.9337	16.17	0.507	0.9751
	308	147.270	0.020	127.263	0.9458	0.35	17.512	0.9133	23.17	0.676	0.9404
	318	192.430	0.030	198.861	0.9801	0.39	29.272	0.9570	41.20	0.405	0.9934
Pb^2+^	298	58.797	0.051	103.438	0.9768	0.76	8.8254	0.9696	29.08	0.169	0.9488
	308	63.659	0.050	115.161	0.9897	0.60	15.226	0.9849	29.73	0.444	0.8860
	318	68.923	0.045	121.942	0.9948	0.59	16.971	0.9922	31.19	0.463	0.9178
Zn^2+^	298	47.569	0.018	45.776	0.9425	0.35	6.874	0.9207	8.646	0.244	0.9482
	308	50.769	0.016	51.868	0.9712	0.35	6.584	0.9593	9.885	0.205	0.9837
	318	54.520	0.013	62.727	0.9509	0.37	7.950	0.9339	11.44	0.182	0.9612

**Table 4:** Adsorption thermodynamic parameters of Cr^6+^, Cu^2+^, Pb^2+^, and Zn^2+^ onto magnetic nano-chitosan in aqueous solution.

Ions	ΔG (kJ/mol)			ΔH (kJ/mol)	ΔS (J/(mol·K))
	298K	308K	318K		
Cr^6+^	−5.959	−6.166	−6.598	3.53	31.701
Cu^2+^	−1.243	−1.734	−2.883	23.099	81.302
Pb^2+^	−3.422	−3.941	−4.233	8.709	40.805
Zn^2+^	−1.949	−2.175	−2.572	7.317	30.986

**Table 5:** Isothermal model fitting parameters for Langmuir and Freundlich.

Ions	Competition Experiment	q_exp_ (µmol/g)	Langmuir			Freundlich		
			K_L_ (L/µmol)	q_m_ (µmol/g)	R^2^	1/n	K_F_ (µmol/g)/(µmol/L)^1/n^	R^2^
Cu^2+^	Single	97.584	0.0156	102.644	0.9096	0.304	17.125	0.9881
	Cu^2+^-Pb^2+^	81.959	0.0057	89.400	0.9353	0.480	4.433	0.9479
	Cu^2+^-Zn^2+^	92.272	0.0037	119.390	0.9991	0.520	4.724	0.9838
	Ternary	79.684	0.0229	78.4616	0.8818	0.287	11.3541	0.9971
Pb^2+^	Single	62.637	0.0034	250.406	0.9989	0.874	1.458	0.9996
	Pb^2+^-Cu^2+^	42.415	0.0023	145.305	0.9993	0.841	0.618	0.9990
	Pb^2+^-Zn^2+^	43.864	0.0043	106.041	0.9969	0.742	1.264	0.9902
	Ternary	35.474	0.0140	60.205	0.9369	0.647	3.109	0.8975
Zn^2+^	Single	47.566	0.0173	48.938	0.9045	0.257	9.848	0.9316
	Zn^2+^-Cu^2+^	44.182	0.0014	53.965	0.9507	0.603	0.969	0.9755
	Zn^2+^-Pb^2+^	26.157	0.0025	39.558	0.9648	0.478	1.053	0.9891
	Ternary	41.372	0.0011	30.659	0.8201	0.6268	1.022	0.8705

## Data Availability

All the data have been included in the study.

## Sample Availability

Samples of the compounds are not available from the authors.
